# Loureirin B suppresses RANKL-induced osteoclastogenesis and ovariectomized osteoporosis via attenuating NFATc1 and ROS activities

**DOI:** 10.7150/thno.35414

**Published:** 2019-07-03

**Authors:** Yuhao Liu, Chao Wang, Gang Wang, Youqiang Sun, Zhangrong Deng, Leilei Chen, Kai Chen, Jennifer Tickner, Jacob Kenny, Dezhi Song, Qingwen Zhang, Haibin Wang, Zhenqiu Chen, Chi Zhou, Wei He, Jiake Xu

**Affiliations:** 1 Department of Joint Orthopaedic, the First Affiliated Hospital, Guangzhou University of Chinese Medicine, Guangzhou, Guangdong 510405, China; 2 School of Biomedical Sciences, University of Western Australia, Perth, WA 6009, Australia; 3 The Lab of Orthopaedics of Chinese Medicine of Lingnan Medical Research Center, Guangzhou University of Chinese Medicine, Guangzhou, Guangdong 510405, China; 4 The First Clinical Medical College, Guangzhou University of Chinese Medicine, Guangzhou, Guangdong 510405, China; 5 Research Centre for Regenerative Medicine, Guangxi Medical University, Nanning, Guangxi 530021, China

**Keywords:** Loureirin B, Nuclear factor of activated T cells 1, Reactive oxygen species, Osteoclast, Osteoporosis

## Abstract

**Rationale**: Osteoporosis is a severe bone disorder that is a threat to our aging population. Excessive osteoclast formation and bone resorption lead to changes in trabecular bone volume and architecture, leaving the bones vulnerable to fracture. Therapeutic approaches of inhibiting osteoclastogenesis and bone resorption have been proven to be an efficient approach to prevent osteoporosis. In our study, we have demonstrated for the first time that Loureirin B (LrB) inhibits ovariectomized osteoporosis and explored its underlying mechanisms of action *in vitro*. **Methods**: We examined the effects of LrB on RANKL-induced osteoclast differentiation and bone resorption, and its impacts on RANKL-induced NFATc1 activation, calcium oscillations and reactive oxygen species (ROS) production in osteoclasts* in vitro*. We assessed the *in vivo* efficacy of LrB using an ovariectomy (OVX)-induced osteoporosis model, which was analyzed using micro-computed tomography (micro-CT) and bone histomorphometry. **Results**: We found that LrB represses osteoclastogenesis, bone resorption, F-actin belts formation, osteoclast specific gene expressions, ROS activity and calcium oscillations through preventing NFATc1 translocation and expression as well as affecting MAPK-NFAT signaling pathways *in vitro*. Our *in vivo* study indicated that LrB prevents OVX-induced osteoporosis and preserves bone volume by repressing osteoclast activity and function. **Conclusions**: Our findings confirm that LrB can attenuate osteoclast formation and OVX-induced osteoporosis. This novel and exciting discovery could pave the way for the development of LrB as a potential therapeutic treatment for osteoporosis.

## Introduction

Bone is a complex tissue that provides support and protection for soft tissues, regulates mineral homeostasis, and maintains the microenvironment of the medullary cavity 1, 2. Several bone disorders, including osteoporosis, are the results of changes in trabecular bone volume and architecture leading to bone fragility fractures 3, 4.

The bone remodeling process is coordinated by several types of cells, including the bone lining cells, osteoclasts, osteoblasts and osteocytes 5, 6. Osteoclasts, which differentiate from the macrophage lineage, are responsible for bone resorption 7. Osteoclastogenesis is a complex process that requires multiple regulators. Initial studies found that osteoclasts formed when bone marrow cells were co-cultured with bone marrow stromal cells 8. Later studies identified that myeloid hematopoietic precursors fused together to form multinucleated osteoclasts under the influence of two specific cytokines: macrophage colony stimulating factor (M-CSF), interacting with receptor c-fms, and receptor activator of NF-κB ligand (RANKL) 9, 10. M-CSF and RANKL are both required for osteoclast differentiation. Excessive production of these cytokines results in increased osteoclast differentiation and abnormal bone resorption, which leads to bone mass loss in osteoporosis 11. It has been reported that increasing the level of reactive oxygen species (ROS) in osteoclasts may promote osteoclast formation and activation 12-14. In addition, an increase in ROS production has been implicated in pathological bone resorption associated with estrogen deficiency and inflammatory arthritis 15-17.

Nuclear factor of activated T cells (NFAT) is a transcription factor first identified in activated T cells and consists of several members: NFATc1, NFATc2, NFATc3, NFATc4 and NFAT5 18. Within these family members, NFATc1 is regulated by the calcium oscillation signaling pathway 19. During osteoclastogenesis, NFATc1 was reported to be auto-amplified and to regulate osteoclast differentiation 20. NFATc1 was identified to be induced by RANKL stimulation 21. Several signaling pathways are involved in calcineurin-mediated dephosphorylation leading to NFAT activation 21. NF-κB and c-Fos pathways can enhance NFATc1 expression by RANKL stimulation. The activation of NFATc1 drives increasing expression via its autoregulatory mechanism.

NFATc1 was also identified to be a master regulator of osteoclastogenesis *in vivo*. It has been reported that NFATc1-deficiency precursor cells cannot rescue the *in-vivo* osteopetrosis phenotype due to failure to differentiate into osteoclasts 20. In addition, embryonically lethal NFATc1 knockout mice were rescued by intracardiac expression of NFATc1 and the rescued mice showed a severe osteopetrosis at birth 22. NFATc1-deleted mice developed a serious osteopetrosis due to the increasing of bone mass and failure to degrade primary spongiosa with resulting calcified cartilage accumulation 23.

*Sanguis draxonis*, also known as Dragon's Blood, is a Chinese traditional herb that has been used against diabetes 24. It has been reported that *Sanguis draxonis* contains more than 12 kinds of active compounds and has been used in anti-AIDS-related diarrhea 25. Loureirin B (LrB) is an active component isolated from *Sanguis draxonis* and has been widely used as a therapy for blood stasis, oxidative stress, cancers, inflammatory conditions and immune disorders 26. Previous studies showed that LrB has the biological effects on anti-algogenesis 27, 28 and promoting insulin secretion 29, 30. LrB was also reported to be an inhibitor of fibrosis through MAPK pathway 31, 32.

Given the significant role of osteoclasts in osteoporosis, and the anti-inflammatory, antioxidant and other applications of LrB, we hypothesized that LrB might suppress osteoclast activity, thus preventing osteoporosis. In the present research, we focused on the potential therapeutic effects of LrB on RANKL-induced osteoclast activity *in vitro* and an ovariectomy (OVX)-induced osteoporosis mouse model *in vivo*, and evaluated the effect of LrB on ROS, NFATc1 and MAPK pathways to elucidate the underlying mechanisms.

## Methods

### *In vitro* osteoclastogenesis assay

Fresh bone marrow macrophages (BMMs) were isolated from C57BL/6 mice using the methods approved by University of Western Australia Animal Ethics Committee (RA/3/100/1244) as described 33, 34, and grown in culture medium (25 ng/ml of M-CSF, 100U/ml of Penicillin/Streptomycin and 10% FBS in α-MEM) in T75 flasks. After the cells were confluent they were removed from the flask using Tryple reagent (Thermofisher, Scoresby, Australia) and scraping, and then seeded into a 96-well plate at 6×10^3^ cells per well with culture medium overnight. The next day, BMMs were stimulated with RANKL at the concentration of 50 ng/ml and the presence of LrB or other compounds, and then medium replaced every two days until osteoclasts formed. After 5 days the cells were then fixed with 2.5% glutaraldehyde in phosphate-buffered saline (PBS) for 10 minutes and stained for tartrate-resistant acidic phosphatase (TRAcP) activity. TRAcP positive multinucleated cells (MNCs) were scored as osteoclast-like (OCL) cells if they had three or more nuclei.

### MTS assay for cell proliferation and viability

Cell proliferation was assessed using a commercially available MTS assay kit (Promega, Sydney, Australia, Cat# 234180). BMMs were seeded at 6×10^3^ cells per well in a 96-well plate and incubated with culture medium overnight. Different concentrations of LrB (or other compounds) were added to each well and then incubated for 48 hours. MTS solution (20 μl/well) was then added to each well for two hours. The effect of compounds on cells was measured by absorbance at 490 nm using a spectrophotometer (BMG, Germany).

### Immunofluorescence staining of F-actin belts and NFATc1 activity

BMMs were seeded in 35 mm glass bottom microwell dishes at the concentration of 6×10^3^ cells per well and cultured with stimulating medium (50 ng/ml of RANKL, 25 ng/ml of M-CSF, 100 U/ml of P/S and 10% FBS in α-MEM) in presence or absence of LrB. After 5 days of stimulation, cells were fixed with 4% paraformaldehyde (PFA) for 10 minutes. After fixation, cells were washed with PBS three times and permeabilized with 0.1% Triton X-100 for 5 minutes. Fixed cells were blocked with 3% BSA-PBS and stained with Rhodamine-Phalloidin (Invitrogen, USA, Cat# 899165) for 1.5 hours. NFATc1 protein expression and localization was detected using a primary NFATc1 antibody (Santa Cruz, USA, Cat# G3014). Cells were incubated with primary antibody for 2 hours and then incubated with Alexa Fluor-488 (Invitrogen, USA, Lot# 185348) conjugated secondary antibody (Sigma Aldrich, Australia, Cat# 97M6809V). Cell nuclei were then stained with Hoechst 33258 (Thermo Fisher, USA, Cat# 1884373) for 10 minutes. Cells were washed with PBS three times and mounted in Prolong Gold antifade mounting medium (Thermo Fisher, USA, Cat#1847311) for confocal microscopy imaging (Nikon A1S confocal microscopy, Japan). F-actin size and nucleus number were measured using ImageJ software (NIH, Bethesda, MD).

### RNA isolation and Real-Time PCR analysis of gene expression

BMMs were seeded in a 6-well plate (1×10^5^ cells per well) and cultured with stimulating medium in the presence or absence of LrB for 5 days to form osteoclasts. Total RNA was isolated from cells using Trizol reagent according to the manufacturer's protocol (Thermo Fisher, Australia, Lot# 180506). cDNA was generated from RNA samples using M-MLV reverse transcriptase and oligo dT primers (Promega). The qPCR efficiency was calculated using diluted cDNA. Polymerase chain reaction amplification for osteoclast specific sequences was then performed. The PCR cycling parameters used were: 94℃ for 5 minutes, 40 cycles of 94℃ for 40 seconds, then 60℃ for 40 seconds and 72℃ for 40 seconds, the final extension step was 5 minutes at 72℃. The following primers were used for detecting specific gene expressions: Acp5 (Forward: 5'-TGTGGCCATCTTTATG CT-3'; Reverse: 5'-GTCATTTCTTTGGGGCTT-3'), *Atp6v0d2* (Forward: 5′-GTGAGACCTTGGAAGACCTGAA-3′; Reverse: 5′-GAGAAATGTGCTCAGGGGCT-3′), *Ctsk* (Forward: 5'-GGGAGAAAAACCTGA AGC-3'; Reverse: 5'-ATTCTGGGGACTCAGAGC-3'), *Mmp9* (Forward: 5′-CGTGTCTG GAGATTCGACTTGA-3′; Reverse: 5′- TTGGAAACTCACACGCCAGA-3′), *c-fos* (Forward: 5′- GCGAGCAACTGAGAAGAC-3′; Reverse: 5′- TTGAAACCCGAGAACATC- 3′), *Ctr* (Forward: 5′-TGGTTGAGGTTGTGCCCA-3′; Reverse: 5′- CTCGTGGGTTTGCCTCATC-3′), and *Hprt* (Forward: 5'-CAGTCCCAGCGTCGTGATTA-3'; Reverse: 5'-TGGCCTCCCATCTCCTTCAT-3') was used as a housekeeping gene. All gene expression results were measured using a ViiA™ 7 Real-time PCR machine (Applied Biosystems, United Kingdom). The relative expression for each target gene was measured using the comparative 2^-ΔΔCT^ method.

### Transfection of RAW cells with ARE vector

To evaluate the effect of LrB on activation of osteoclastic intracellular ROS-related transcription factors, antioxidant response element (ARE) luciferase vector was transfected in to RAW264.7 cells. RAW264.7 cells were seeded into a 24-well plate and cultured overnight in DMEM containing 10% FBS and P/S (100U/ml) to adhere. Cells were then transfected with pGL4.37 [luc2P/ARE/Hygro] Vector (Promega, Australia, LOT# 0000271030) according to the Lipofectamine^TM^ 3000 reagent protocol (Invitrogen, Australia). 12 hours after transfection, the medium was changed to DMEM containing 15% FBS. Transiently transfected cells were seeded in a 24-well plate for further investigation to measure Nrf2-ARE activity.

### Luciferase reporter assays of NF-κB, NFATc1 and Nrf2-ARE

The RAW264.7 cell line (ATCC, Manassas, Virginia, USA) was stably transfected with NF-κB 35 and NFATc1 luciferase reporter gene constructs 36, and transiently with Nrf2-ARE luciferase reporter gene construct, and then seeded in 48-well plates at the concentration of 1.5×10^5^, 5×10^4^ and 1×10^5^ cells per well respectively. Cells were cultured overnight and then pre-treated with LrB for 1 hour and stimulated with RANKL for 6, 24 and 12 hours respectively. After stimulation, cells were lysed using luciferase lysis buffer and luciferase activities were measured using a luciferase reporter assay kit (Promega, Sydney, NSW, Australia, Cat# 318248) and a luminescence plate reader (BMG LABTECH, Ortenberg, Germany).

### Western blot analysis

Freshly isolated BMMs were seeded in 6-well plates at the concentration of 1x10^5^ cells per well. The cells were stimulated with RANKL on day 0, 1, 3 and 5 in the presence of LrB at 10 μM. Cells were harvested after treatment and lysed with RIPA lysis buffer (50 mM Tris-HCl pH7.5, 150 mM NaCl, 1% Nonidet P-40, 0.1% SDS, 1% sodium deoxycholate). For the short-term Western Blotting assay, BMMs were seeded in 6-well plates and cultured with culture medium until they reached 90% confluence. LrB was used to pretreat the cells for 1 hour followed by 0, 5, 10, 20, 30 and 60 minutes of RANKL stimulation. Total cellular proteins were extracted using RIPA lysis buffer. SDS-PAGE was used to separate proteins, and the protein bands were transferred to a nitrocellulose membrane.

After 2 hours of blocking with 5% skim milk, NFATc1, c-fos (Cell Signaling, USA, Cat# 2250S) CTSK, V-ATPase-d2 (Santa Cruz, USA, Cat# C0810 and L1415), p-JNK (R&D System, USA, Cat# MAB1205), p-p38 (Cell Signaling, USA, Cat# 4511L) and IκB-α, p-ERK (Santa Cruz, USA, Cat# C1313 and D1117) primary antibodies were added onto the membranes and then incubated overnight at 4°C. The corresponding secondary antibodies were then administrated and incubated for 2 hours. Antibody reaction was detected using Western Lightning Ultra Detection Kit (PerkinElmer, USA, Lot#204-18251) and images were taken by the FujiFilm LAS-4000 Gel Documentation System (Tokyo, Japan) and its associated software.

### Hydroxyapatite resorption assay

In order to detect osteoclast activity, BMMs (seeded at 1×10^5^ cells per well) were stimulated with 50 ng/ml RANKL and 25 ng/ml M-CSF to form osteoclasts in 6-well collagen-coated plates. When osteoclasts were formed, cells were detached gently using cell dissociation solution (Sigma Aldrich, Australia, Cat# SLBT 0287) and seeded into 96-well hydroxyapatite plates (Corning, USA, Lot#31417018) in equal numbers. Mature osteoclasts were incubated in stimulating medium with or without LrB treatment at concentrations of 5 μM and 10 μM. 48 hours later, half of the wells were stained to identify OCL cells in each well. In the remaining wells, cells were bleached and discarded. The images of hydroxyapatite coating wells were captured using a Nikon microscope (Nikon Corporation, Minato, Tokyo, Japan) and pits of resorption areas were measured using Image J software (NIH, Bethesda, Maryland, USA). The resorbed area per well and the percentage of resorbed area per osteoclast were used to quantify the osteoclast activity.

### Measurement of intracellular ROS activities

Intracellular ROS activity was investigated using 6-carboxy-2', 7'-dichlorodihydrofluorescein diacetate (carboxy-H_2_DCFDA) dye according to the manufacturer's protocol (Molecular Probes, Australia, Lot# 1756365). BMMs were seeded in 35 mm glass bottom microwell dishes at the concentration of 6×10^3^ cells per well. After cells were adherent, the medium was replaced with stimulating medium containing LrB for 48 hours. Cells were starved for one hour in Hanks Balanced salt solution (HBSS). The HBSS was replaced by carboxy-H_2_DCFDA staining solution (carboxy-H_2_DCFDA was dissolved in HBSS at the concentration of 20 μM) and incubated for 30 minutes at 37 ℃. The staining solution was changed to HBSS after incubation and cells were incubated for 2 minutes to avoid temperature change. Intracellular ROS activity was measured by inverted A1Si confocal microscope. Fluorescence intensities were captured and measured using an NIS-Elements Viewer software.

### Measurement of intracellular Ca^2+^ oscillation

Intracellular Ca^2+^ oscillation was measured using a Fluo4-AM dye in accordance with the manufacturer's protocol. BMMs were seeded into 48-well plates and cultured in culture medium overnight. The following day cells were treated with LrB and stimulated with 50 ng/ml RANKL overnight. Cells were washed with washing buffer (HBSS containing 1mM probenecid and 1% FBS) and then incubated with 5 μM Fluo4 staining solution (Fluo4-AM in 20% Pluronic-F127 (w/v)) for 45 minutes. After incubation, cells were washed once and incubated at room temperature for 20 minutes followed by another two washes. Cells were visualized with an inverted fluorescence microscope (Nikon Eclipse Ti, Japan) every 2 seconds for 3 minutes and intracellular calcium oscillation was observed and marked. Oscillation intensity was calculated by the difference of maximum and minimum fluorescence intensities.

### Ovariectomy (OVX)-induced osteoporosis mouse model

All *in vivo* experiments were approved by the Institutional Animal Ethics Committee of the First Affiliated Hospital, Guangzhou University of Chinese Medicine (Ethic No. SYL2018002). Twenty-four C57BL/6J mice (females; 18.6 ± 1.4 g, 11 weeks old) were supplied by the Animal Experiment Center of the First Affiliated Hospital, Guangzhou University of Chinese Medicine. All mice were randomly divided into three groups: sham group (n=8), OVX group (n=8), and OVX+LrB group (n=8). Bilateral ovariectomies were performed to induce osteoporosis under chloral hydrate anesthesia for OVX and OVX+LrB groups. For sham group, the ovaries were only exteriorized but not resected. All mice had 5 days recovery after the operations, then an intraperitoneal injection of LrB (4 mg/kg, every 2 days for 6 weeks) was delivered for OVX +LrB group. The sham and OVX group mice were intraperitoneally injected with PBS as a vehicle control.

### Micro-CT, bone histomorphometry and gene expression level analysis

After sacrificing the experimental mouse groups, right femurs (n=8 for each treatment group) were fixed with 4% PFA for 24 hours and placed in 1.5 ml microcentrifuge tubes and scanned using Skyscan 1176 micro-CT scanner (Bruker micro-CT, Kontich, Belgium). The scanning was carried out using following settings: voltage, 50 kV; source current, 500 μA; Al 0.5 mm filter; pixel size 9 μm; rotation step, 0.4 degree. For trabecular bone analysis, a region of interest (0.5 mm above the growth plate on distal femur with a height of 1 mm) was selected. The bone volume/tissue volume (BV/TV), trabecular number (Tb.N), trabecular thickness (Tb.Th) and trabecular separation (Tb.Sp) were measured using CT Analyser program (Bruker micro-CT, Kontich, Belgium). Two- and three- dimensional images were generated using Data-viewer and CTvol softwares (Bruker micro-CT, Kontich, Belgium) respectively.

Following micro-CT analysis, all femurs were decalcified in 14% EDTA (Sigma-Aldrich, Australia, Cat# BCBW0411) at 37 ℃ for 7 days. Femurs were then processed through ethanol and xylene into wax, embedded into paraffin blocks and sectioned on a microtome at a thickness of 5µm. Hematoxylin and eosin (H&E) and TRAcP staining were performed. Images for each section were taken by Aperio Scanscope (Mt Waverley, VIC, Australia) and bone histomorphometric analysis was performed using BIOQUANT OSTEO software (Bioquant Image Analysis Corporation, Nashville, TN, USA).

Left femurs were collected and total RNA was isolated using Trizol reagent, and total protein was acquired using RIPA lysis buffer. *Ctsk* and *Atp6v0d2* gene expressions were determined by qPCR, V-ATPase-d2 and CTSK protein expression levels were measured by Western Blotting as described above.

### Statistical analysis

All data and statistical analysis were followed with the recommendation of pharmacology experimental design^37^. All experimental data was collected from triplicate experiments and presented as the mean ± SD and statistical significance was determined by one-way or two-way ANOVA. A possibility level of p-value < 0.05 was considered as statistically significant.

## Materials and Reagents

Six compounds (Loureirin [Lr] A, LrB, LrC, LrD, Cochinchinenin [Cc] A and CcC) extracted from Dragon's Blood resin were purchased from Chengdu Must Bio-Technology Co., Ltd (Chengdu, Sichuan Province, China) and dissolved in nuclease-free water with DMSO. Alpha modified Eagles Medium (α-MEM, Lot# 1897009), DMEM (Cat# 1896968), HBSS and fetal bovine serum (FBS) were obtained from Gibco (Sydney, Australia). Recombinant RANKL was obtained as previously reported 38, and recombinant M-CSF was purchased from Sigma Aldrich (Australia, Cat#M6518). Penicillin/streptomycin was purchased from Sigma Aldrich (USA, Cat# 076M4762V).

## Results

### LrB inhibits osteoclastogenesis and actin belt formation

**Figure 1:**
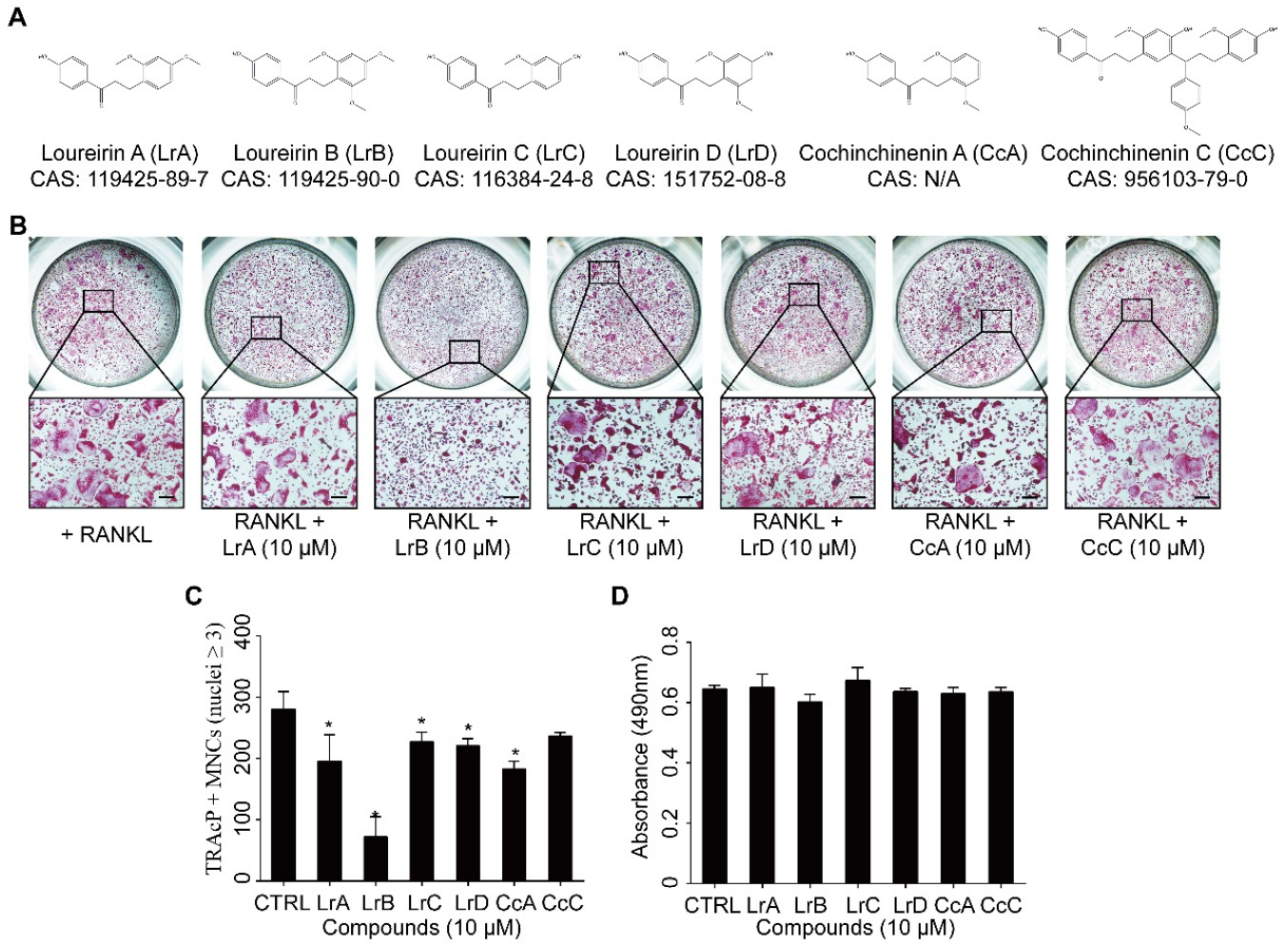
** Compounds extracted from Dragon's Blood inhibit RANKL-induced osteoclast formation with no cytotoxicity *in vitro*. (A)** The molecular structure and CAS number of each compound. **(B)** BMMs were seeded into a 96-well plate at a density of 6x10^3^ cells/well and stimulated with in the presence or absence of Dragon's Blood compounds for 5 days respectively. TRAcP staining was performed until osteoclast formed. Representative images of TRAcP-positive cells showed that all six compounds (10 μM) can suppress osteoclast formation to some extent, and LrB exhibited the best inhibitory function. **(C)** Quantification analysis of TRAcP-positive multinucleated cells (MNCs, nuclei ≥3). **(D)** BMMs (6x10^3^ cells/well) were cultured with 25 ng/ml of M-CSF in the presence or absence of Dragon's Blood compounds (10 μM) for 2 days respectively, then the cytotoxic effect of compound on BMMs was measured by an MTS assay. All bar charts are presented as mean ± SD; n=3; Scale bar=200μm. *p<0.05, relative to non-treatment group.

**Figure 2:**
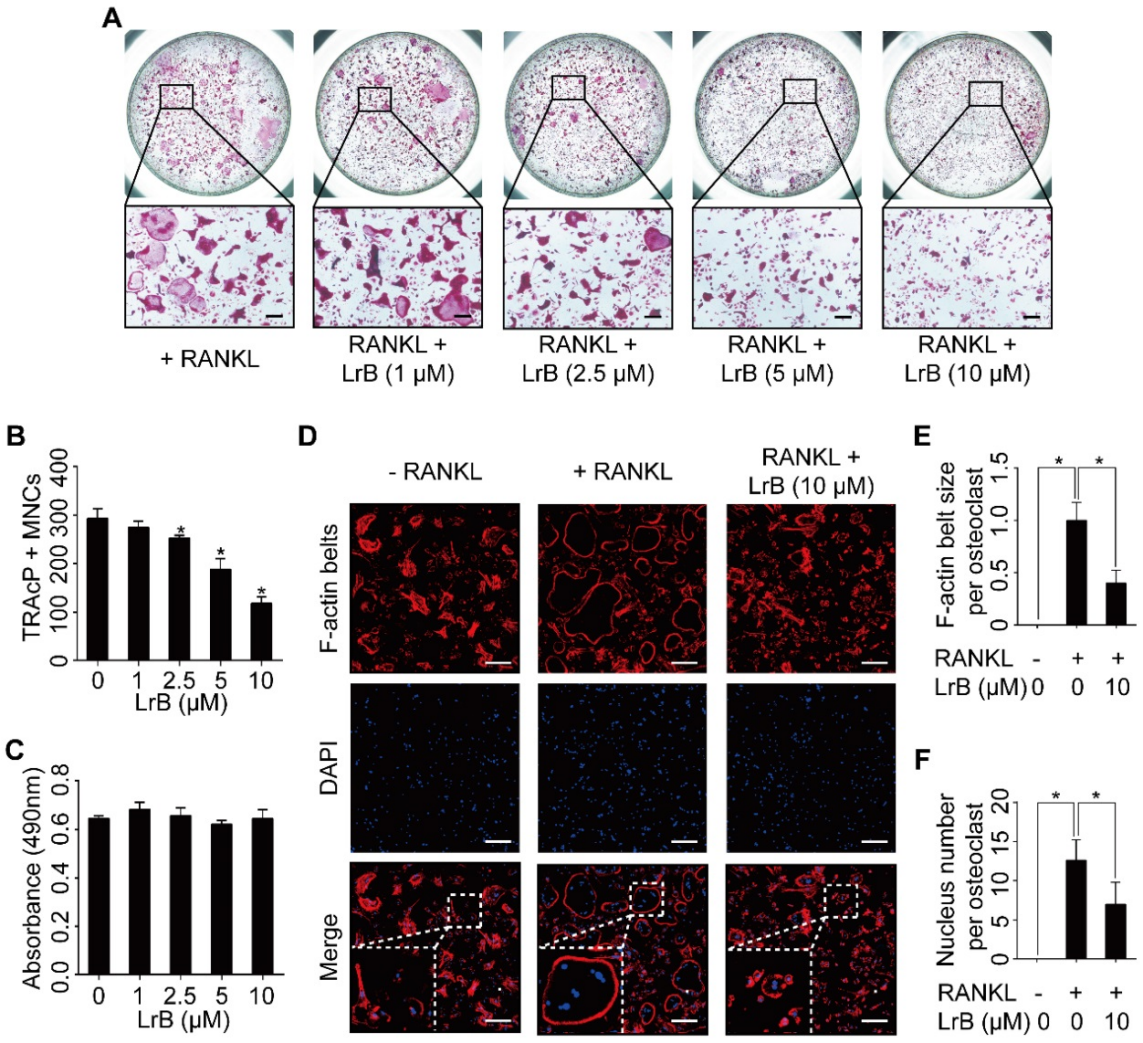
** LrB attenuates RANKL-induced osteoclastogenesis and actin ring formation. (A)** BMMs were stimulated with 25 ng/ml of M-CSF and 50 ng/ml of RANKL in the presence or absence of LrB with concentrations ranging from 1μM to 10 μM for 5 days, and then TRAcP staining was performed. **(B)** Quantification analyses of osteoclastic cells indicated that LrB started to inhibit osteoclast formation at a concentration of 2.5 μM. **(C)** The cytotoxic effect of various concentrations of LrB on BMMs after 48 hours as detected by an MTS assay. **(D)** BMMs were plated into a 96-well plate and stimulated with 25 ng/ml of M-CSF and 50 ng/ml of RANKL in the presence or absence of indicated concentration of LrB for 5 days. Cells were fixed with 4% PFA for 10 minutes, permeabilized with 0.1% Triton X-100 for 5 minutes and stained with Rhodamine-Phalloidin (1.5 hours) and DAPI (10 minutes) subsequently. Representative images of actin belts formation were observed with confocal microscopy, and F-actin belts were stained as Red, and nuclei stained as Blue. **(E-F)** Quantification analyses of F-actin size and nucleus number per osteoclast. All bar charts are presented as mean ± SD; n=3; Scale bar=200μm. *p<0.05, relative to non-treatment group.

To investigate the effect of Dragon's Blood compounds on osteoclastogenesis, an initial screen was performed. BMMs were stimulated with RANKL and M-CSF with 10 μM of LrA, LrB, LrC, LrD, CcA and CcC to form osteoclasts. Among all these compounds, LrB showed the most significant inhibition of osteoclastogenesis without detectable cytotoxic effect (**Figure 1**). To further investigate the effect of LrB on osteoclast formation, a dose dependent effect of LrB was examined during RANKL-induced osteoclast formation. Increasing concentrations of LrB inhibited TRAcP-positive osteoclast formation (**Figure 2A and 2B**). In addition, a cell proliferation assay was performed to identify whether the inhibition effect of LrB on osteoclasts was cytotoxic. The results indicate that concentrations of LrB ranging from 1 μM to 10 μM were not cytotoxic to BMMs (**Figure 2C**). We next examined osteoclast size and nuclearity through staining F-actin belts and nuclei which were found to be significantly suppressed by LrB at concentrations of 5 μM and 10 μM (**Figure 2D, 2E and 2F**).

We then performed an osteoclastogenesis time-course analysis which showed a dramatic inhibition of osteoclast formation after treatment with LrB during the early stage of osteoclast formation (**Figure S1A, S1B and S1C**).

These data show that LrB inhibits RANKL-induced osteoclastogenesis and F-actin belt formation without causing cytotoxic ^effects^.

### LrB impairs hydroxyapatite resorption and represses osteoclast specific gene expressions

**Figure 3:**
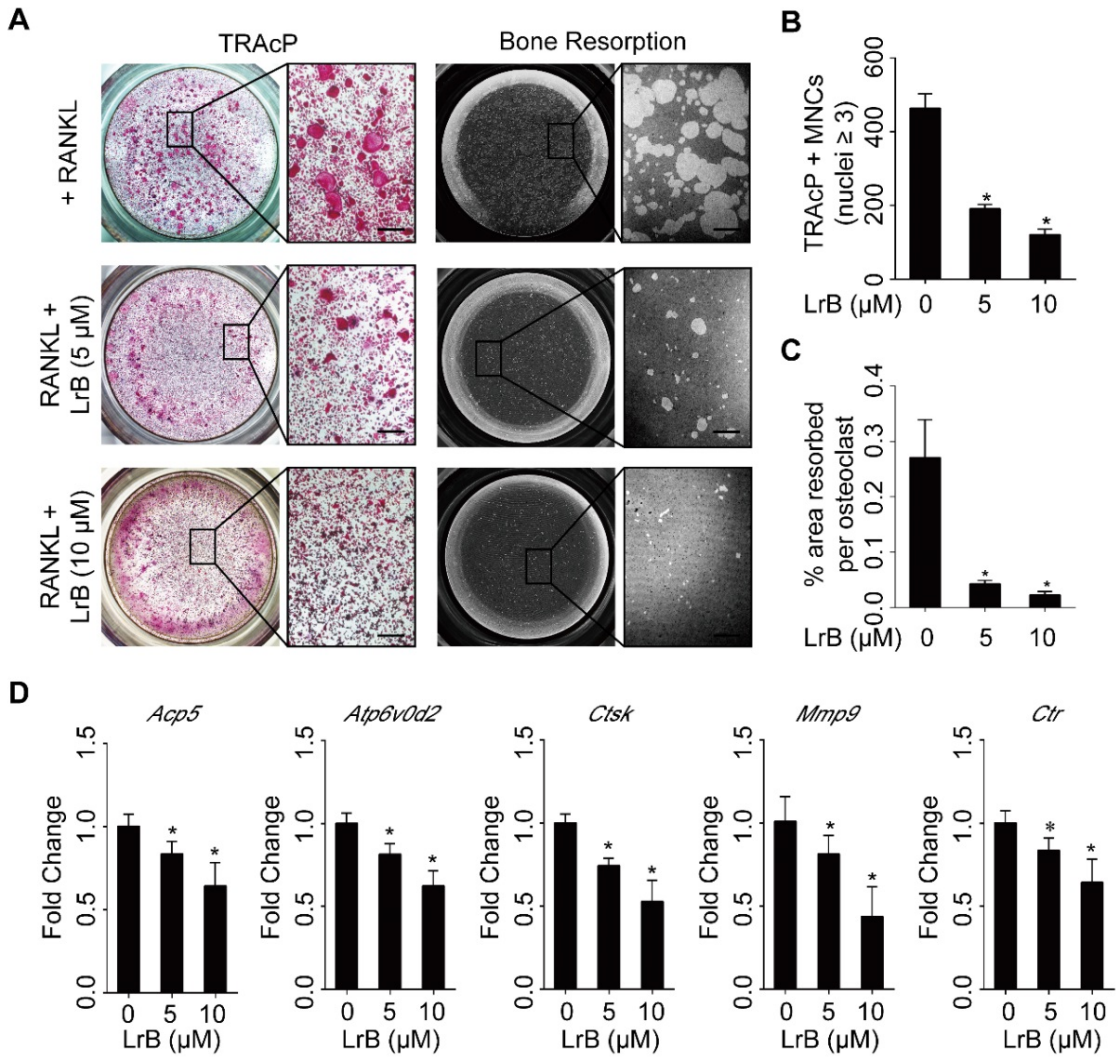
** LrB impairs osteoclastic resorption ability and gene expression. (A)** BMMs, plated into one 6-well collagen-coated plate at 1x10^5^ cells/well, were stimulated with 25 ng/ml of M-CSF and 50 ng/ml of RANKL until osteoclast began to form. Then osteoclast precursors were transferred into one 96-well hydroxyapatite plate, and incubated with indicated concentrations of LrB until mature osteoclasts were generated. Half of wells in each group were stained with TRAcP solution and the remaining wells were bleached for observing resorptive area. **(B-C)** Quantification analyses of osteoclast number each well and resorbed area per cell. **(D)** BMMs were seeded into one 6-well plate at 1x10^5^ cells/well and stimulated with 25 ng/ml of M-CSF and 50 ng/ml of RANKL in the presence or absence of LrB with different concentrations for 5 days. RNA was isolated from each group for synthesizing cDNA and performing RT-qPCR, and *Acp5*, *Atp6v0d2*, *Ctsk*, *MMP9* and *Ctr* were detected. All bar charts are presented as mean ± SD; n=3; Scale bar=200μm. *p<0.05, relative to non-treatment group.

To identify the effect of LrB on osteoclast activity, a hydroxyapatite resorption assay was performed. Consistent with the lack of cytotoxicity observed in BMMs, the number of mature osteoclasts was not affected following LrB treatment (**Figure 3A and 3B**). However, the resorbed areas per osteoclast were significantly repressed by LrB (**Figure 3A and 3C**).

The efficiency of real time PCR was 96.33%, and the results showed that osteoclast specific gene expressions, including *Acp5*, *Atp6v0d2, Ctsk*,* Mmp9* and* Ctr* were significantly up-regulated during osteoclast differentiation, but suppressed dose-dependently with LrB treatment (**Figure 3D**).

### LrB represses RANKL-induced activation of MAPK and NFATc1 pathways

**Figure 4:**
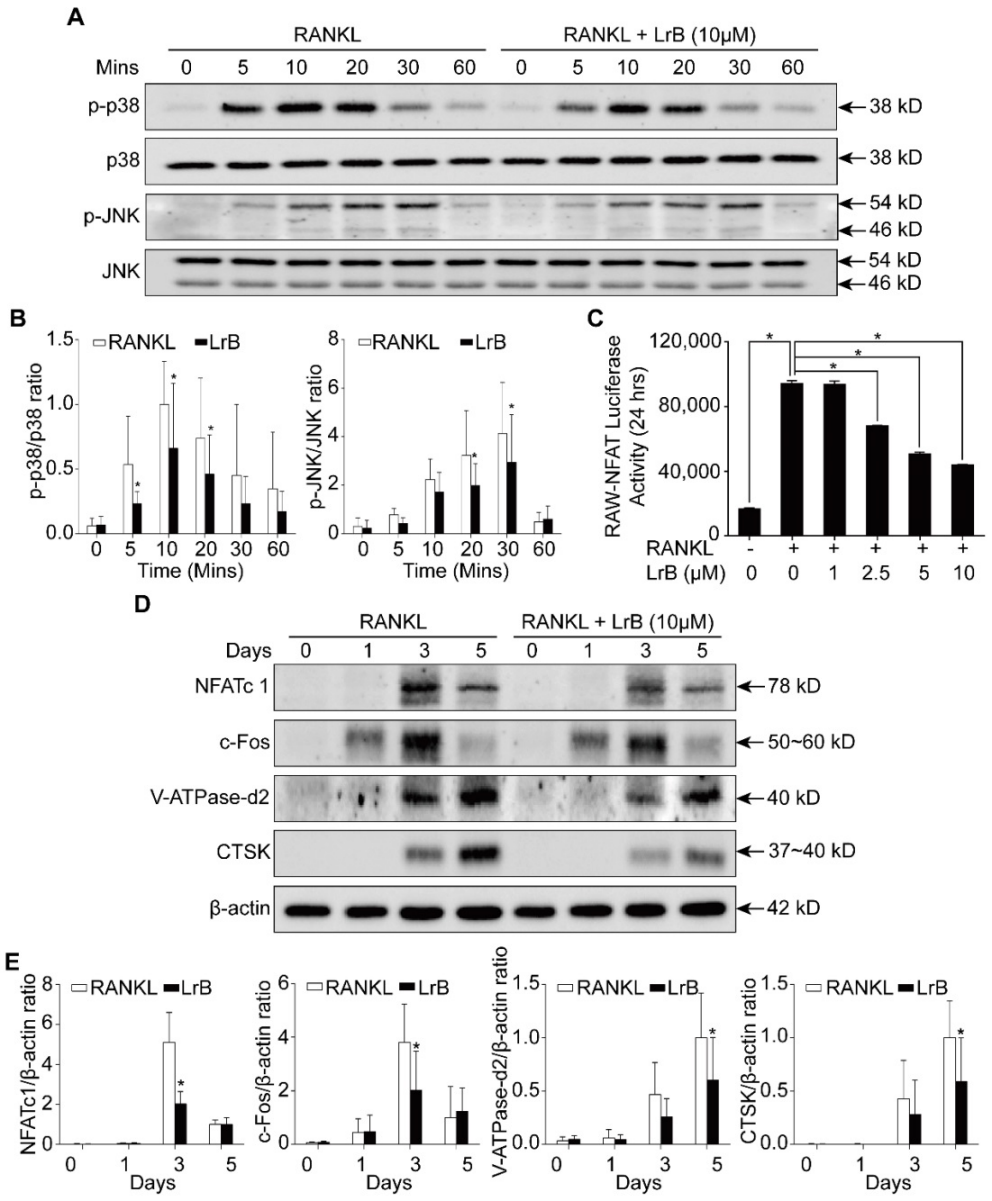
** LrB inhibits MAPK and NFATc1 signaling pathways. (A)** BMMs were seeded in 6-well plates and cultured with culture medium until they reached 90% confluence. LrB was used to pretreat the cells for 1 hour followed by 0, 5, 10, 20, 30 and 60 minutes of RANKL stimulation. Total cellular proteins were extracted using RIPA lysis buffer and cell lysates were analyzed by Western blotting using primary antibodies specific to p-p38 and p38, p-JNK and JNK. Representative images showed the inhibition effect of LrB on MAPK pathway signaling specific to p-p38 and p-JNK. **(B)** Quantitative analyses of p-p38 and p-JNK were normalized to total p38 and JNK respectively. Phosphorylation levels of p38 and JNK were significantly suppressed by LrB from 5 minutes to 30 minutes. **(C)** NFATc1 transcriptional activity was measured by a luciferase reporter gene assay. RAW264.7 cell line was stably transfected with an NFATc1 luciferase reporter gene. RAW-NFAT-luc cells were seeded in 48-well plate and pretreated with LrB with different concentrations for 2 hours, then stimulated by RANKL for 24 hours. Cells were then lysed by lysis buffer and luciferase activity was measured using a luciferase reporter assay kit. Quantitative analyses indicated that LrB inhibits NFATc1 transcriptional activity dose-dependently. **(D)** Freshly isolated BMMs were seeded in 6-well plates at the concentration of 1x10^5^ cells per well. The cells were stimulated with RANKL on day 0, 1, 3 and 5 in the presence or absence of LrB at 10 μM. Primary antibodies including NFATc1, c-Fos, V-ATPase-d2 and CTSK were applied and β-actin was used as a normalized control protein. Representative images were used to indicate the LrB's inhibitory effect on NFATc1 pathway activities. **(E)** Quantification of the ratios of band intensity of NFATc1, c-Fos, V-ATPase-d2 and CTSK relative to β-actin. All bar charts are presented as mean ± SD; n=9. *p<0.05, relative to non-treatment group.

To further investigate the mechanism by which LrB exerts its inhibitory effect on osteoclast differentiation, we examined the impact of LrB on MAPK and NFATc1 pathways. Phosphorylation of three MAPK family members including ERK, JNK and p38 was upregulated by RANKL stimulation. We found that LrB treatment suppressed phosphorylated JNK and p38 kinase after 20 minutes and 5 minutes respectively (**Figure 4A and 4B**), whereas phosphorylation of ERK was not significantly affected (**Figure S2A**). In addition, RANKL-induced IκB-α protein degradation was delayed by LrB (**Figure S2B and S2C**). Further, NF-κB luciferase reporter assay showed that LrB also inhibited RANKL-induced NF-κB activity (**Figure S2D**).

As shown in **Figure 4D and 4E**, RANKL induced NFATc1 protein expression was attenuated by LrB, consistent with its inhibitory effect on osteoclastogenesis. Further, LrB reduced NFATc1 transcriptional activity induced by RANKL in a dose-dependent manner as measured by luciferase reporter gene assay (**Figure 4C**). In addition, LrB also suppressed the protein expression levels of c-fos, V-ATPase-d2 and CTSK, all of which are important for osteoclast formation and function (**Figure 4D and 4E**). Collectively, LrB represses MAPK and NFATc1 activity, thus influencing downstream signaling and transcription.

### LrB interferes with RANKL-induced intracellular calcium oscillation and NFATc1 translocation

**Figure 5:**
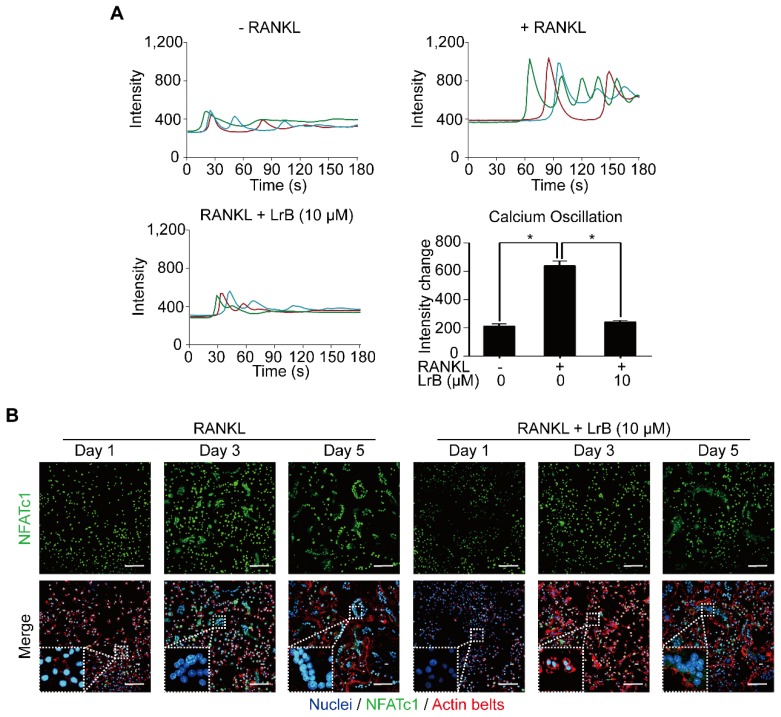
** LrB attenuates calcium oscillation and NFATc1 translocation. (A)** Representative calcium signal fluctuations with different treatment. Average intensity change per cell was measured. Calcium oscillations were analyzed across multiple cells for each treatment, peak and baseline intensity were calculated (n>16 individual cell per well, triplicate wells were applied for each group). **(B)** NFATc1 translocation was presented by immunofluorescence staining. BMMs were treated with LrB (10 μM) and stimulated by RANKL (50 ng/ml) for required days, and then 4% PFA fixed cells were blocked by 3% BSA-PBS and stained with Rhodamine-Phalloidin for 1.5 hours. For detecting NFATc1 activity, primary NFATc1 antibody was used to incubate the cells for 2 hours and then reacted with Alexa Fluro-488 conjugated second antibody. The Hoechst 33258 was used to stain the cell nucleus. Images were taken by confocal microscopy to observe **NFATc1**, **nuclei** and **F-actin**. All bar charts are presented as mean ± SD; n=3; Scale bar=100μm. *p<0.05, relative to non-treatment group.

With RANKL stimulation, the activation of calcium transduction pathway initiates calcium oscillations, which induces NFATc1 self-amplification and nuclear translocation. The effect of LrB on the calcium pathway and NFATc1 translocation were examined and shown in **Figure 5**. As expected, RANKL treatment showed significant calcium oscillation signals compared with the non-induced group. RANKL-induced calcium oscillation signals were attenuated in the LrB treatment group (**Figure 5A**).

Images taken by confocal microscopy demonstrated that LrB reduces the time-dependent (Day 1, Day 3 and Day 5) RANKL-induced NFATc1 nuclear translocation, especially at Day 3 (**Figure 5B**), consistent with its inhibitory effect on osteoclast formation.

### LrB attenuates RANKL-induced ROS production in BMMs

**Figure 6:**
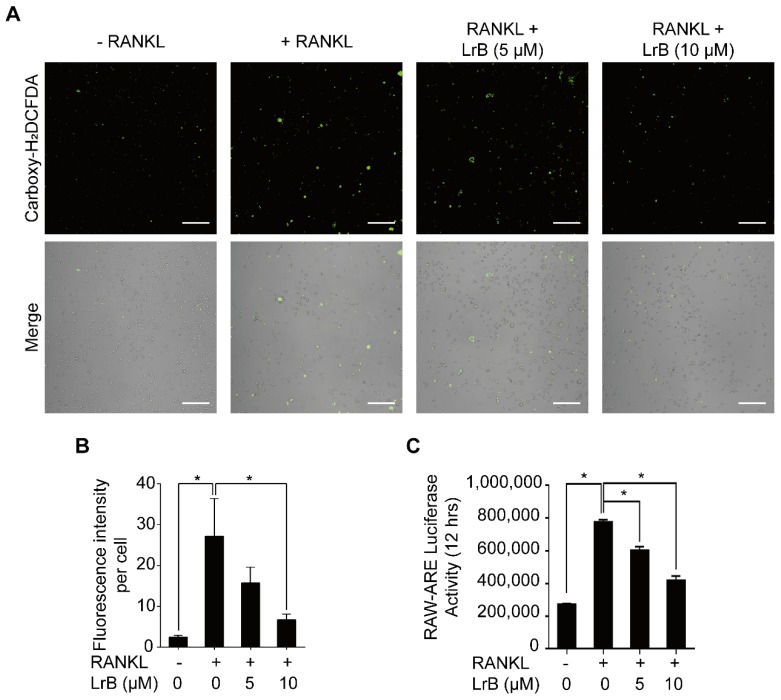
** LrB suppresses intracellular ROS activity. (A)** Representative confocal images of RANKL-induced ROS production in the presence or absence of pre-treated LrB. Intracellular ROS was detected by a carboxy-H2DCFDA dye in the form of highly fluorescent DCF. The lower panel is a merge of DCF fluorescence and confocal digital interference contrast images. **(B)** Quantification of DCF fluorescence intensity in an average per cell. **(C)** Oxidative stress was indicated by ARE transcriptional activity and measured by luciferase reporter gene. All bar charts are presented as mean ± SD; n=3; Scale bar=200μm. *p<0.05, relative to non-treatment group.

To investigate the effect of LrB on RANKL-induced intracellular ROS levels during osteoclast differentiation, oxidation-sensitive dye carboxy-H_2_DCFDA was used to visualize the oxidative fluorescent signals using confocal microscopy. The results showed that the fluorescence intensity of the LrB treatment group was significantly decreased in a dose-dependent manner compared with the RANKL treatment group (**Figure 6A and 6B**).

Furthermore, the effect of LrB on ROS-mediated ARE transcriptional activity was investigated by a luciferase reporter gene assay. With RANKL stimulation, ROS -mediated ARE activity was increased at 12 hours. Consistent with the observed reduction in ROS levels, ARE activity was remarkably down-regulated in the presence of LrB (**Figure 6C)**, indicating that ROS production was effectively eliminated by LrB. Therefore, LrB suppresses osteoclast formation via inhibiting ROS production.

### LrB protects against OVX-induced bone loss

**Figure 7:**
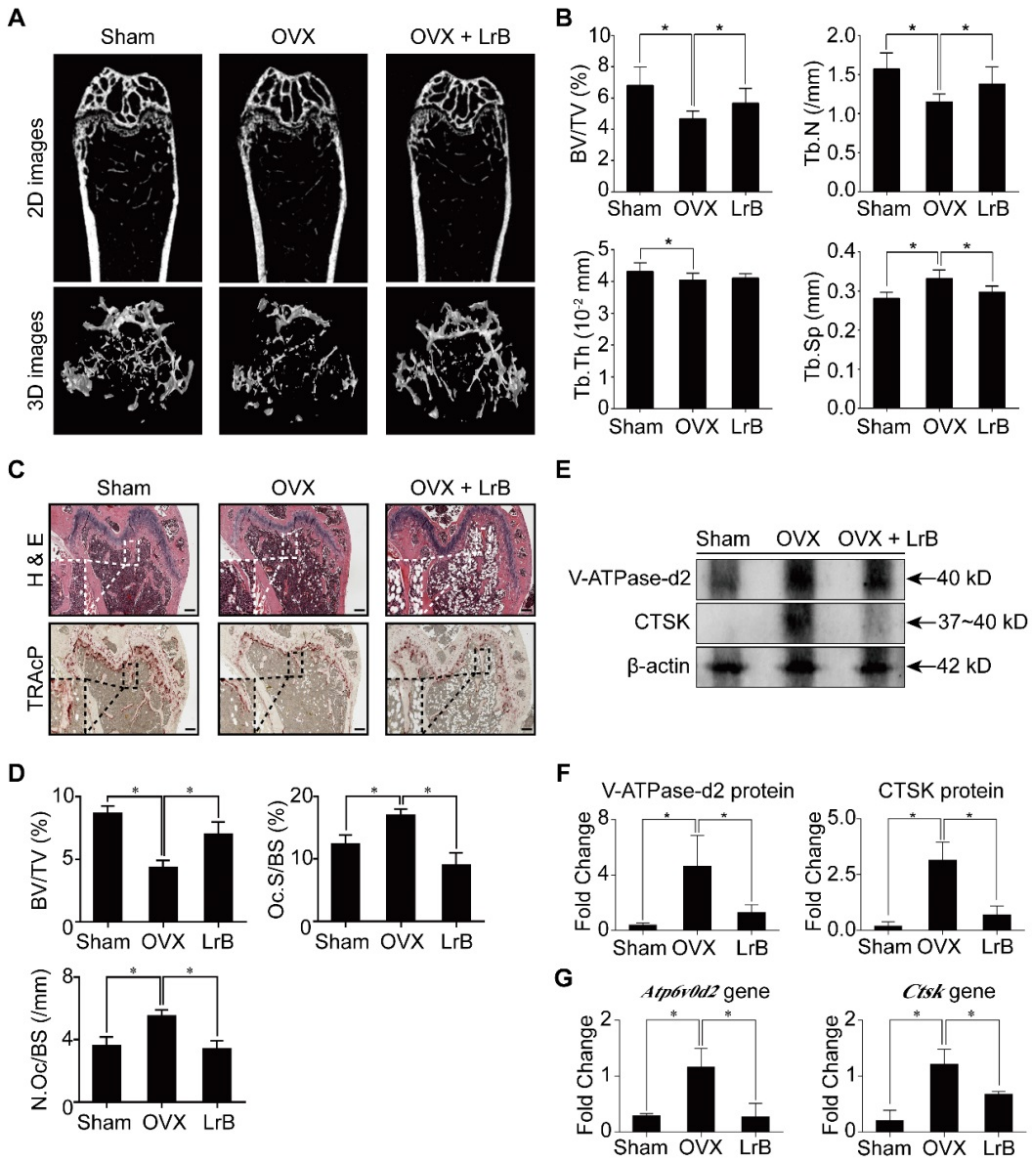
** LrB prevents OVX-induced bone mass loss *in vivo*.** All mice were randomly divided into three groups: sham group (n=8), OVX group (n=8), and OVX+LrB (4 mg/kg) group (n=8). Bilateral ovariectomy was performed to induce osteoporosis under avertin (250 mg/kg, i.p) anesthesia in OVX and OVX+LrB groups. For the mice of sham group, the ovaries were only exteriorized but not resected. All mice had 5 days recovery after the operations, then an intraperitoneal injection of LrB (4 mg/kg every 2 days for 6 weeks) was performed for the mice in the OVX +LrB group. The sham and OVX group mice were intraperitoneally injected with PBS as a vehicle control. **(A)** Representative Micro-CT images of 2D and 3D demonstrating that OVX-induced bone loss was prevented by LrB treatment. **(B)** Quantitative analyses of parameters regarding to bone architecture, including BV/TV, Tb.N, Tb.Th, Tb.Sp (n=8). **(C)** Representative images of H&E, TRAcP staining of decalcified bone sections. **(D)** Quantitative analyses of all bone sections, including BV/TV, Oc.S/BS and N.Oc/BS (n=6). **(E-F)** V-ATPase-d2 and CTSK protein expressions in bone tissue (n=3). **(G)** Gene expressions of Atp6v0d2 and Ctsk in bone tissue (n=3). All bar charts are presented as mean±SD; Scale bar=500μm. *p<0.05, relative to OVX group. H&E, hematoxylin and eosin; TRAcP, tartrate resistant acid phosphatase; BV/TV, bone volume per tissue volume; Tb.N, trabecular number; Tb.Th, trabecular thickness; Tb.Sp, trabecular separation; Oc.S/BS, osteoclast surface/bone surface; N.Oc/BS, osteoclast number/bone surface.

Having established that LrB has an effect on inhibiting osteoclast formation and bone resorption, we then investigated the potential of LrB as a prophylactic agent to prevent OVX-induced osteoporosis *in vivo*. Mice were OVX- or sham- operated and then injected with LrB (4mg/kg) every 2 days, or vehicle for 6 weeks post-surgery. After the OVX procedure and LrB treatment, there were no adverse events or fatalities recorded. Furthermore, body weights were not significantly affected by LrB or vehicle injection (**Figure S3**). Micro-CT analysis showed that the LrB prevented the extensive bone mass loss in the OVX mouse model. Quantitative analysis confirmed that bone parameters, including BV/TV, Tb.N and Tb.Sp, were increased in the LrB treatment group (**Figure 7A and 7B**). Histological analysis further confirmed that OVX-induced bone mass loss was significantly reduced by the LrB treatment when compared with the non-treatment group. Quantification of H&E staining indicated that the bone surface and bone volume were well maintained in the LrB treatment group. TRAcP staining showed the osteoclast number per bone surface and osteoclast surface area per bone surface were decreased after LrB treatment when compared with the non-treatment group (**Figure 7C and 7D**). Total RNA and protein were isolated from the femurs of each treatment group. As shown in **Figure 7E, 7F, and 7G**, the osteoclast marker genes *Ctsk* and *Atp6v0d2,* which are responsible for osteoclast function, were suppressed by LrB compared with the non-treatment group both in gene expressions and protein levels.

## Discussion

Bone tissue is constantly remodeled to maintain skeletal homeostasis throughout our lifespan. This biological process is tightly regulated by two main cell types: osteoclasts and osteoblasts through their coupling activities 6, 39. Osteoclasts, which are giant multinucleated cells formed from the macrophage lineages, are responsible for resorbing bone and releasing mineral matrix 40. In contrast, osteoblasts are differentiated from mesenchymal stem cells and they play a major role in bone formation 41. The delicate balance between resorption and formation of bone tissues is essential for healthy skeletal growth and maintenance. However, as aging progresses, increased osteoclastic bone resorption leads to the deterioration of bone structures, mass and integrity. The excessive osteoclast activity leads to severe osteoporosis and current clinical therapies are mainly focused on estrogen replacement, bisphosphonates or Denosumab. These applications are effective but also have long term side-effects including potential risk of breast cancer, and atypical femur facture 42, 43. Therefore, searching for novel alternative drugs may pave the way to improve the treatment of osteoporosis. In this study, we demonstrated for the first time that LrB inhibits osteoclastogenesis by repressing ROS, MAPK and NFATc1 activities* in vitro* and prevents the development of OVX-induced osteoporosis mouse model *in vivo*.

Firstly, to evaluate the biological function of LrB, an osteoclast differentiation assay was carried out. It was revealed that LrB significantly inhibited osteoclast differentiation in a dose-dependent manner. F-actin belts were stained and visualized indicating that LrB interferes the podosome belt formation, which further confirmed the inhibitory effect of LrB on osteoclast formation. Hydroxyapatite resorption assays demonstrated that LrB suppresses osteoclastic resorption, indicating that the effect of LrB was on osteoclast differentiation and resorbing function.

Accumulating evidence indicates that MAPK family members, including ERKs, JNKs, and p38, are closely involved in RANKL-induced osteoclast differentiation 44. With RANKL stimulation, ERK, JNK and p38 are phosphorylated. JNK and p38 are more related to osteoclastogenesis while ERK is crucial for osteoclast survival 45-47. In the present study, Western Blot results indicated that LrB attenuated the phosphorylation of JNK and p38 without affecting ERK, suggesting that LrB suppressed osteoclastogenesis but not osteoclast survival.

NFATc1 has been reported as the dominating transcriptional regulator of osteoclast differentiation, also well-known is its self-amplification to maintain robust expression. Several lines of evidence show the critical role of NFATc1 in osteoclast formation and function. Specifically, lack of NFATc1 leads to the failure to form osteoclasts from embryonic stem cells, NFATc1 disruption in hematopoietic cells results in increased bone mass and decreased osteoclasts in a mouse model 21, 23. The results of our research showed that the expression level and transcriptional activity of NFATc1 following RANKL stimulation were repressed by LrB. Furthermore, osteoclast specific genes, including *c-fos*, *Atp6v0d2* and *Ctsk,* which are all regulated by NFATc1 directly 21, 48, were suppressed by LrB. NFATc1 is also essential in the release of intracellular calcium during osteoclastogenesis. With RANKL stimulation, intracellular calcium oscillation is induced which continuously activates calcineurin and triggers NFATc1 activation and its auto-amplification 21. We found that LrB could suppress the intensity of Ca^2+^ oscillation in response to RANKL stimulation, and RANKL-induced NFATc1 nuclear translocation was also blocked in the presence of LrB during osteoclast formation, consistent with the pivotal role of calcium signaling in NFATc1 stimulation.

Further investigation into the mechanisms revealed that LrB also affects osteoclast activity through the regulation of ROS. Accumulating evidence indicates that ROS may also regulate the activity of key osteoclast transcription factors such as NF-κB. It has been reported that ROS influences the activation of NF-κB by disturbing the phosphorylation of IκBα 12. Conversely, NF-κB can regulate ROS activity by enhancing the production of antioxidant enzymes 49. Another critical osteoclast transcription factor, NFATc1, is also associated with the activity of ROS. ROS activity is known to be generated by RANKL-induced stimulation which also induces Ca^2+^ oscillation, leading to the upregulation and auto-amplification of NFATc1 50. In our current study, we demonstrated that NFATc1 transcriptional activity was suppressed by LrB. However, it is unclear whether the regulation of NF-κB and NFATc1 by LrB is dependent on ROS, which requires further investigation. ARE is a downstream factor of nuclear factor-erythroid 2-related factor 2 (Nrf2) which regulates the expression of many antioxidant enzymes 51. Under common conditions, Nrf2 is bound to actin fibers in the cytoplasm with Keap1 and degraded by proteasomes following activation. After RANKL stimulation, Nrf2 is exposed to oxidative stress which leads to its translocation into nuclei. Nrf2 then heterodimerizes with Maf protein and binds together with ARE. This heterotrimer will activate the transcriptional activation of antioxidant enzyme genes 52. Interestingly, LrB was found to suppress intracellular ROS production during RANKL-induced osteoclastogenesis in our study. Furthermore, ARE transcriptional activity was down-regulated by LrB indicating that LrB scavenges ROS in the cytoplasm, indicative of a key role of LrB in eliminating ROS in osteoclasts.

Based on these *in vitro* results, we established an OVX mouse model to further investigate whether LrB has potential therapeutic effect *in vivo*. We can conclude that LrB exhibits a remarkable protective effect on OVX-induced bone loss in a mouse model as confirmed by micro-CT and H&E staining. Moreover, osteoclast formation and function were reduced by LrB treatment, which is consistent with the *in vitro* study. In addition, *Ctsk* and *Atp6v0d2* expressions, genes which are responsible for bone resorption, were down-regulated *in vivo* in the LrB treatment group.

**Figure 8:**
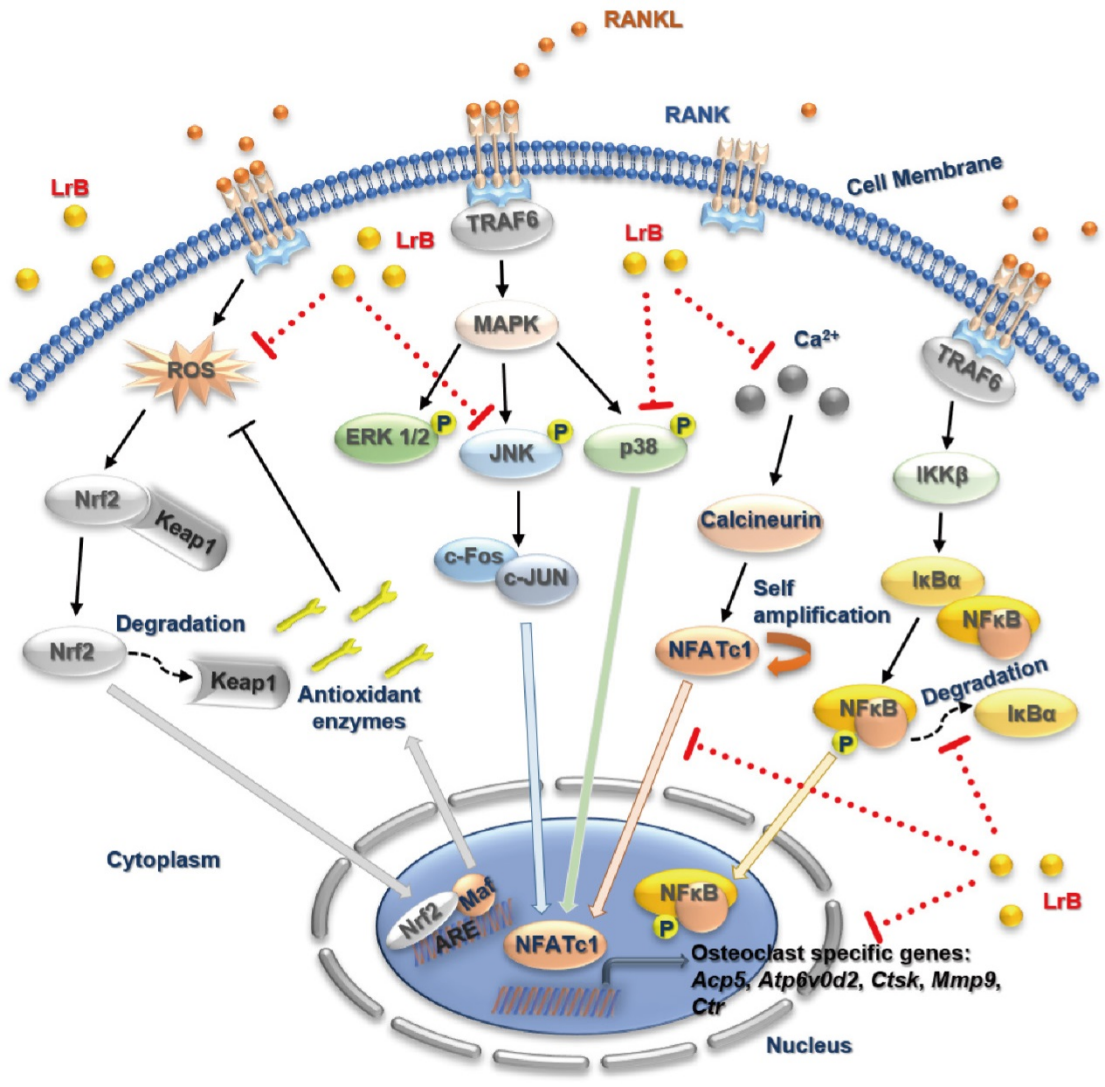
** A proposed working model for the inhibition of LrB on osteoclastogenesis.** Upon RANKL binding with RANK, both MAPK and NF-κB pathways are activated, leading NFATc1 self-amplification and translocation into nucleus. Also, ROS production is enhanced by the stimulation of RANKL. The activation of TRAF6, which in return, results IκBα and Keap1 degradation, calcium oscillation and up-regulation of osteoclast specific genes. Our results indicated that LrB can suppress osteoclast formation and bone resorption via attenuating NFATc1 and ROS activities. Acp5, acid phosphatase 5, tartrate resistant; Atp6v0d2, ATPase H+ Transporting V0 Subunit D2; c-fos, Proto-oncogene C-Fos; Ctsk, cathepsin K; Mmp9, matrix metallopeptidase 9.

In summary, our study has demonstrated for the first time that LrB can inhibit osteoclast formation and function via suppressing ROS, MAPK and NFATc1 activities, which further attenuates downstream osteoclast gene expressions (**Figure 8**). Additionally, LrB was also found to prevent OVX-induced osteoporosis *in vivo* via repressing *Ctsk* and *Atp6v0d2* gene and protein expressions in the bone tissue microenvironment. We also found that LrB of 10μM shows little effect on osteoblastic bone nodule formation (**Figure S4**). In conclusion, these findings could pave the way to the potential development of LrB-targeted therapeutic treatments for skeletal diseases such as osteoporosis.

## Supplementary Material

Supplementary figures and tables.

## Abbreviations

LrB: Loureirin B
BMMs: bone marrow macrophages
FBS: fetal bovine serum
RANKL: receptor activator of nuclear factor‐κB ligand
M-CSF: macrophage-colony stimulating factor
NFATc1: nuclear factor of activated T cells 1
MAPKs: mitogen-activated protein kinases
V-ATPase-d2: ATPase H+ Transporting V0 Subunit D2
c-fos: Protooncogene C-Fos
CTSK: cathepsin K
JNK: c-Jun N-terminal kinase
ERK: extracellular signal-regulated kinase
NF-κB: nuclear factor-κB
PBS: phosphate buffered saline
PCR: polymerase chain reaction
TRAcP: tartrate resistant acid phosphatase
OVX: ovariectomized
ROS: reactive oxygen species
BV/TV: bone volume per tissue volume
Tb.N: trabecular number
Tb.Th: trabecular thickness
DAPI: 4,6-diamidino-2-phenylindole
