# Supplementary material for: Loureirin B suppresses RANKL-induced osteoclastogenesis and ovariectomized osteoporosis via attenuating NFATc1 and ROS activities
Source: Theranostics. 2019 Jul 3;9(16):4648–62. doi: 10.7150/thno.35414 (PMC6643439; doi:10.7150/thno.35414)
Supplement: Supplementary file 1 — Supplementary figures and tables. [file thnov09p4648s1.pdf]

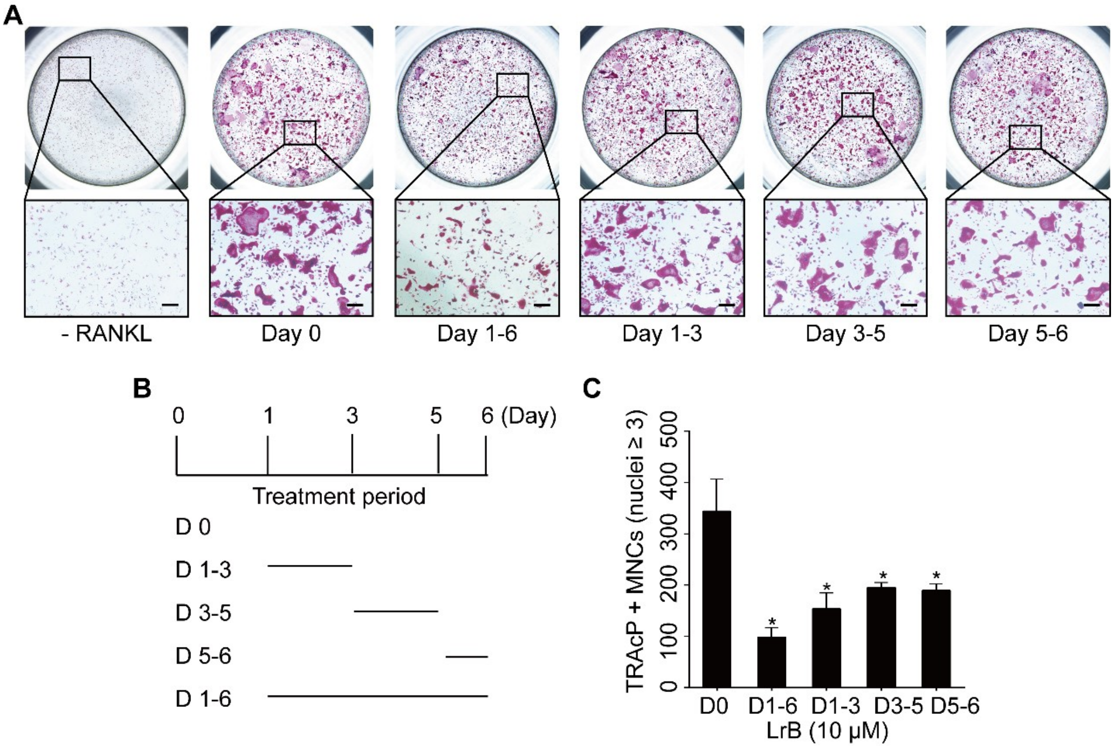

Figure S1. LrB suppresses RANKL-induced osteoclastogenesis at early stage. (A) BMMs were cultured with 25 ng/ml of M-CSF, and 50 ng/ml of RANKL, and LrB (10 μM) were treated at certain time points. TRAcP staining was then performed to observe the osteoclast. TRAcP-positive images were taken for each time course experiment. **(B)** The treatment period was showed as the diagram. **(C)** The number of TRAcP-positive multinucleated cells (MNCs, nuclei ≥3) was counted and analyzed. All bar charts are presented as mean ± SD; n=3. Scale bar=200 μm. **p*<0.05, relative to non-treatment group.

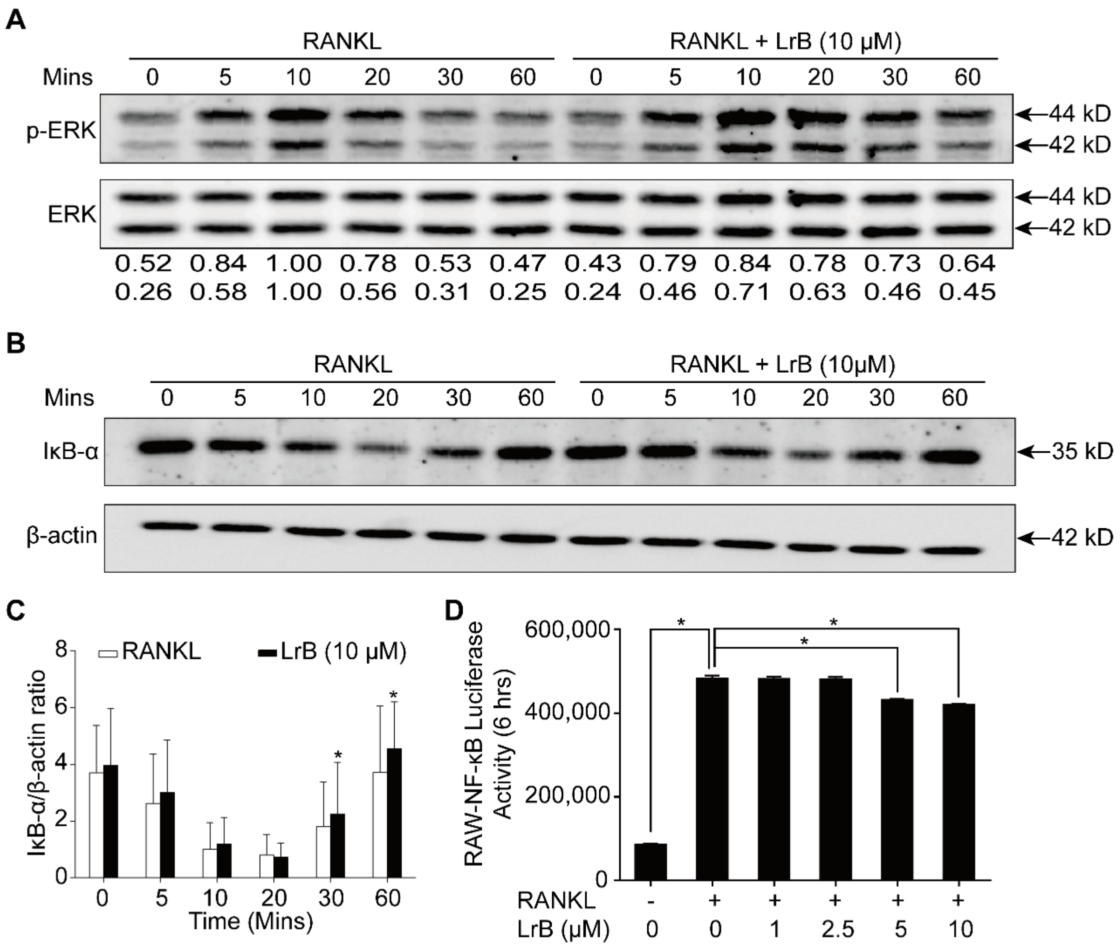

Figure S2. The effect of phosphorylated ERK and NF-κB pathways by LrB. **(A)** Western blotting was performed to detect the effect of LrB on phosphorylation of ERK pathway. Representative images were selected and quantification of p-ERK relative to total ERK was applied. LrB has little effect on p-ERK/MAPK pathway. **(B)** Representative images of LrB upregulated the IκB-α protein expression level from 30 minutes were showed. **(C)** Quantitative analysis was performed. **(D)** NF-κB transcriptional activity was attenuated by LrB. All bar charts are presented as mean ± SD. *$p < 0.05$, relative to non-treatment group.

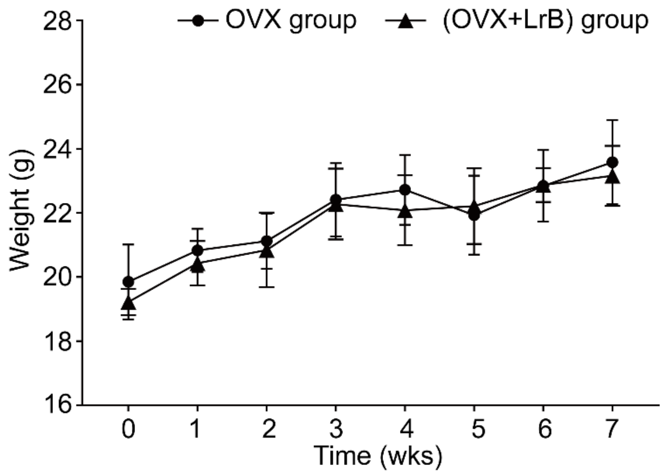

Figure S3. OVX-induced osteoporosis mouse weights were not significantly affected with the LrB or vehicle injection. Bilateral ovariectomy was performed to induce osteoporosis under avertin (250 mg/kg, i.p) anesthesia for OVX and OVX+LrB groups and body weights were measured each week. There are no significant body weight changes observed from each group.

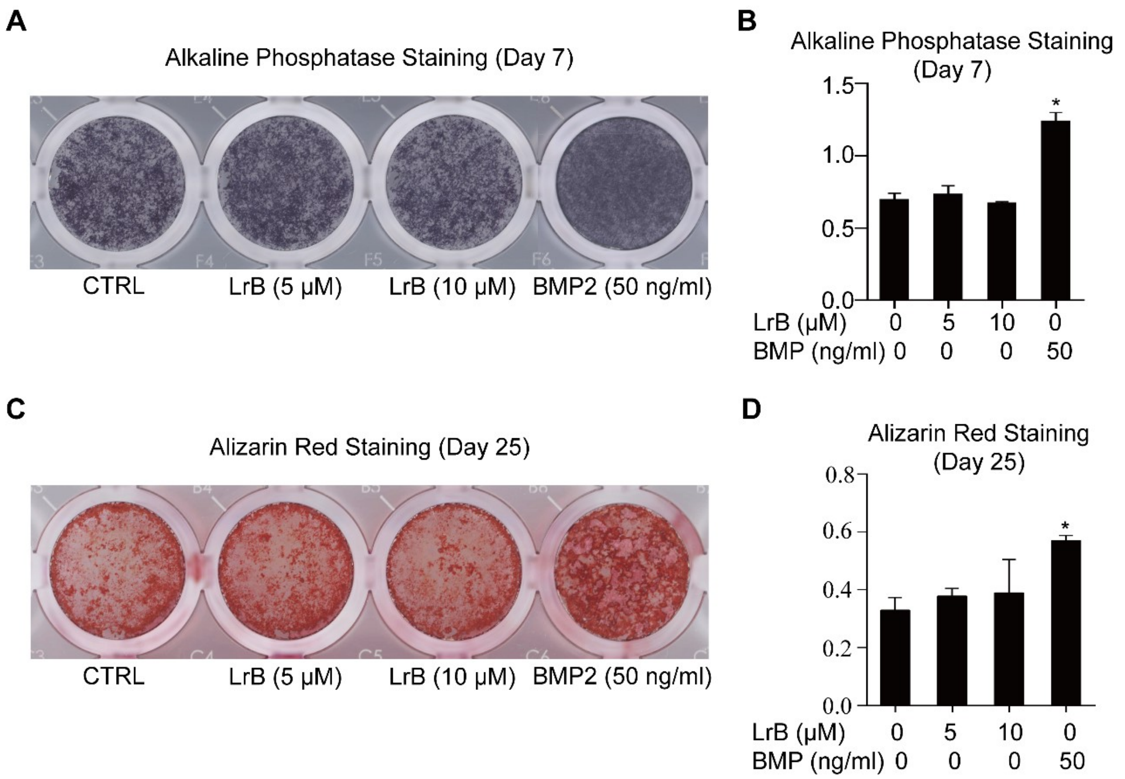

Figure S4. LrB has little effect on osteoblast. (A, C) Primary osteoblasts were isolated from long bones of 8-week-old mice and cultured with D-MEM contained 15% FBS, 1% Glutamax and 1% P/S in T25 flasks, then seeded and cultured in 48-well plates until 90% confluence. 50 μg/ml L-ascorbic acid, 5 mM β-glycerophosphate and 10nm dexamethasone were used to induce osteogenic. After 7 days and 25 days, alkaline phosphatase activity and mineralization activity were measured using alkaline phosphatase staining and alizarin red staining respectively. Representative images were used to indicate the effect of LrB on osteoblast ALP and mineralization activities. **(B, D)** ALP and mineralization positive areas were measured using Image J software respectively and then calibrated. All bar charts are presented as mean ± SD; n=3. *$p < 0.05$, relative to control group.
